# Adherence to Medication among Parkinson's Disease Patients Using the Adherence to Refills and Medications Scale

**DOI:** 10.1155/2022/6741280

**Published:** 2022-02-23

**Authors:** Branislava Radojević, Nataša T. Dragašević-Mišković, Andona Milovanović, Marina Svetel, Igor Petrović, Maja Pešić, Aleksandra Tomić, Dejana Stanisavljević, Miroslav M. Savić, Vladimir S. Kostić

**Affiliations:** ^1^Special Hospital for Cerebrovascular Disorders “Saint Sava”, Belgrade, Serbia; ^2^Clinic of Neurology, School of Medicine, University of Belgrade, Belgrade, Serbia; ^3^Institute for Medical Statistics and Informatics, Faculty of Medicine, University of Belgrade, Belgrade, Serbia; ^4^Faculty of Pharmacy, University of Belgrade, Belgrade, Serbia

## Abstract

**Objectives:**

Adherence to medication is an important factor that can influence Parkinson's disease (PD) control. We aimed to explore patients' adherence to antiparkinsonian medication and determine factors that might affect adherence to medications among PD patients.

**Methods:**

A cross-sectional, exploratory survey of PD patients treated with at least one antiparkinsonian drug and with a total score of MoCA (Montreal Cognitive Assessment) ≥26 was conducted. The final sample included 112 PD patients. A patient's adherence was assessed through ARMS (Adherence to Refills and Medications Scale). ARMS scores higher than 12 were assumed lower adherence. In addition, each patient underwent neurological examination, assessment of depression, anxiety, and evaluation of the presence of PD nonmotor symptoms.

**Results:**

The mean ARDS value in our cohort was 14.9 ± 2.5. Most PD patients (74.1%) reported lower adherence to their medication. Participants in the lower adherence group were younger at PD onset, had significantly higher UPDRS (Unified PD Rating Scale) scores, as well as UPDRS III and UPDRS IV subscores, HARS (Hamilton Anxiety Rating Scale), and NMSQuest (Non-Motor Symptoms Questionnaire for PD) scores compared to the fully adherent group (*p*=0.013, *p*=0.017, *p*=0.041, *p*=0.043, and *p*=0.023, respectively). Among nonmotor PD symptoms, the presence of cardiovascular, apathy/attention-deficit/memory disorders, hallucinations/delusions, and problems regarding changes in weight, diplopia, or sweating were associated with lower adherence. Multivariate regression analysis revealed depression as the strongest independent predictor of lower adherence.

**Conclusion:**

Depressed PD patients compared to PD patients without clinical depression had a three times higher risk for lower adherence to pharmacotherapy. Recognition and adequate treatment of depression might result in improved adherence.

## 1. Introduction

Pharmacological management of PD is multifaceted, and this complexity may negatively impact patients' adherence to therapy [1]. First, motor features (i.e., bradykinesia, tremor, rigidity, and postural instability) differentially respond to levodopa therapy. Second, with the progression of PD, it is usually necessary to increase the frequency of levodopa dosing from three to four and five times a day at a set time, with the addition of a long-acting preparation at bedtime. Also, combinations of antiparkinsonian drugs are more commonly applied, and serious adverse effects are more likely to occur. Thus, therapy aims to be tailored for each patient based on their response and ability to tolerate drugs. Thirdly, PD's nonmotor symptoms (NMSs) encompass neuropsychiatric manifestations (anxiety, apathy, depression, hallucinations, psychosis, and dementia), sleep disorders, dysautonomia, pain, and alteration of smell or vision usually poorly respond to levodopa therapy and require additional medication. Patients in moderate to advanced stages of PD can take up to 5 different drugs distributed over up to 8 intakes [2]. Finally, PD is more common in elderly patients who often have multiple comorbidities, which increases the number of administered medicaments from several drug classes and complicates dosing and titration schedules [3].

Suboptimal adherence is defined as taking less than 80% of the prescribed therapy [4]. The incidence of suboptimal adherence in PD varies between 10% and 67% [5–7]. Such variability, at least partly, maybe due to different methodological approaches since no method is considered the “gold standard” [8]. There are two main methodological approaches for the assessment of adherence: (a) objective (prescription refill rates, residual pill counting, and electronic measurement devices) and (b) subjective method (patient self-reports, family interviews, and questionnaires). Suboptimal adherence in PD is associated with poor control of symptoms, poor outcomes, and higher overall healthcare costs as well as with unnecessary therapy adjustments, such as increasing the doses or introduction of additional drugs [4]. Also, it can produce diagnostic uncertainty and point clinicians to an erroneous conclusion about atypical Parkinsonism conditions [7].

To the best of our knowledge, no studies thus far have assessed adherence to medication among PD patients using the Adherence to Refills and Medications Scale. Our study investigated adherence to therapy in Serbian PD patients and possible predictive factors influencing regular drug intake. This study did not assess social and economic factors and healthcare system barriers. We focused on the impact of patient-, therapy-, and disease-related factors on adherence.

## 2. Patients and Methods

This cross-sectional, exploratory study enrolled over five months (November 2020–March 2021) includes 112 PD consecutive outpatients during regularly scheduled appointments at the Clinic of Neurology, Clinical Centre of Serbia (Belgrade). The local thical committee approved the study. The inclusion criteria were diagnosis of PD according to the United Kingdom Brain Bank criteria, treated with at least one antiparkinsonian drug, and the total score of Montreal Cognitive Assessment (MoCA) ≥26. Also, participants were screened out of the study if a spouse or another individual was involved in reminding them to take their medication. Informed consent was obtained from all individual participants included in the study. Each patient completed a specially constructed questionnaire containing the essential demographic characteristics (sex, age, and level of education), disease-related data (age of onset, duration, and a form of the disease: tremor-predominant or akinetic-rigid), therapy-related data (current therapy, medications used, and doses), and complication-related data (presence of dyskinesias, motor fluctuations, and hallucinations). For all patients, the levodopa equivalent daily dose (LEDD) was calculated according to the standard formula [9]. The severity of parkinsonian signs was evaluated using the Unified PD Rating Scale (UPDRS) [10], while the Hoehn and Yahr PD staging scale (HY) [11] was used to determine the stage of the disease. Daily activities were assessed by the Schwab and England Scale (S&E) [12]. Depression and anxiety were considered by the Hamilton Depression Rating Scale (HDRS) [13] and the Hamilton Anxiety Rating Scale (HARS) [14], respectively. The Non-Motor Symptoms Questionnaire for PD (NMSQuest) [15] was used to evaluate the presence of NMS. Afterward, questions from the NMSQuest were grouped to several relevant domains for further analysis: gastrointestinal tract, urinary tract, sexual function, cardiovascular and falls, apathy/attention/memory, hallucinations/delusions, depression/anxiety/anhedonia, sleep/fatigue, pain unrelated to other causes, and miscellaneous (diplopia, weight loss, and excessive sweating) [15]. Cognition was assessed with the MoCA [16], and patients with cognitive impairment defined as an MoCA score <26/30 were excluded from the study. The Adherence to Refills and Medications Scale (ARMS-12) was used to assess adherence to medication in PD patients [17]. It consists of 12 items, the first eight of which concern the act of taking medications as prescribed, and the other four—the act of filling new prescriptions or refilling prescriptions on time. Each item was structured for a response on a Likert scale with answers of “none,” “some,” “most,” or “all” of the time, which were given values from 1 to 4. Item scores are summed to produce an overall adherence score of 12–48 [17]. ARMS scores higher than 12 were assumed lower adherence [17]. Patients taking more than one antiparkinsonian drug were instructed to answer the questions by thinking about their daily experiences, on average, with all the antiparkinsonian medicines they take, not just a specific medication. Testing was performed during the “on” phase (the phase in which the optimal drug therapy and motor improvement were achieved in the patient).

## 3. Statistical Analyses

Descriptive statistics were used to describe patients' demographic and disease characteristics and medication adherence scores. Categorical data are presented as numbers with percentages. Data were checked for normality using the Kolmogorov–Smirnov test. Differences between groups were analyzed using Mann–Whitney *U* tests for continuous variables and the Pearson chi-squared test for categorical variables. Logistic regression analysis was performed to establish the risk factors for low adherence. Initially, several variables considered necessary for lower adherence were included in a univariate analysis. For HDRS and HARS, as routine diagnostic scores, the widely clinically used cutoffs that differentiate between the normal and pathological status of the patient were applied. The significant risk factors in the univariate analysis were then included in a multivariate model. Results were expressed as B, Wald chi-square, odds ratios (OR), and their 95% confidence intervals (CI). *p* < 0.05 was considered statistically significant. All analyses were conducted using the Statistical Package for the Social Sciences (IBM SPSS, version 21).

## 4. Results

The demographic and clinical characteristics of the patients included in the study are presented in Table 1. Levodopa was the most frequently used drug (108 patients, 96.4%), given in 4.6 ± 0.9 daily doses. Twenty patients (17.9%) were on levodopa monotherapy, while 92 (82.1%) used more than one antiparkinsonian medication. Of the total 77 (68.8%) patients were taking dopamine agonists, 23 (20.5%) were taking amantadine, 4 (3.6%) COMT inhibitors, and 3 (2.7%) MAO-B inhibitors. Of the total number of participants, 38 (33.9%) patients were on antidepressant drugs, 14 (12.5%) used antipsychotics, and 58 (51.8%) were using benzodiazepines. The mean number of used drugs (antiparkinsonian drugs, benzodiazepines, antipsychotics, and antidepressants) per day was 3.3 ± 1.4, a minimum of one and a maximum of seven drugs.

The mean ARMS score was 14.9 ± 2.5; the score ranged from 12 to 22 points. The best possible minimum score was achieved by 29 (25.9%) patients, while 83 (74.1%) patients reported lower adherence to the prescribed therapy. No patient obtained the maximum ARMS score, indicating absolute nonadherence to medication.

ARMS scores were treated as a dichotomized measure, with values of 12 (fully adherent) or >12 (lower adherent) (Table 1). There were no significant differences between the two patient groups regarding sex, age at examination, education level, disease duration, a form of the disease, number of used drugs, number of levodopa daily doses, and presence of dyskinesias. Participants in the lower adherent group were younger at PD onset and used significantly higher average levodopa daily doses and average LEDD than the fully adherent group (*p*=0.022, *p*=0.018, and *p*=0.014, respectively). A significantly higher incidence of motor fluctuations and hallucinations was found in the lower adherence group than the fully adherent group (*p* = 0.022 and *p* = 0.021, respectively). PD patients in the lower adherence group had significantly higher UPDRS scores and UPDRS III and UPDRS IV subscores, HARS, and NMSQuest scores than the fully adherent group (*p*=0.013, *p*=0.017, *p*=0.041, *p*=0.043, and *p*=0.023, respectively). Analysis of domains of the NMSQuest showed a statistically significantly higher number of positive responses in a lower adherent group in the following four domains: cardiovascular, apathy/attention-deficit/memory disorders, hallucinations/delusions, and domain which assessed the presence of changes in weight, excessive sweating, or diplopia (*p*=0.016, *p*=0.026, *p*=0.012, and *p*=0.024, respectively).

The univariate logistic regression analysis demonstrated statistically significant association between lower adherence and following eight variables: age at PD onset (*p*=0.026), levodopa average daily dose (*p*=0.031), LEDD (*p*=0.011), presence of motor fluctuations (*p*=0.024), presence of hallucinations (*p*=0.026), UPDRS total score (*p*=0.044), S&E score (*p*=0.036), HDRS>7 (*p* = 0.011), and NMSQuest total score (*p*=0.025) (Table 2). Multivariate logistic regression analysis revealed that independent predictor for lower adherence in PD were depression (OR = 3.449; 95% CI:1.425–8.350; *p* = 0.006).

## 5. Discussion

In this sample, 74.1% of patients reported lower adherence to their medication schedule (ARMS scores of >12). Compared with those who reported “perfect” adherence (the best possible minimum score measured by ARMS), participants with lower adherence had more severe disease, poorer motor status, more therapy-related complications such as motor fluctuations and hallucinations, poorer daily functioning, higher anxiety scores, and more nonmotor symptoms. The strongest predictor of lower adherence was depression, which increases the risk of lower adherence 3.4 times.

Different studies have used various tools to evaluate and assess PD patients' adherence to medication, including self-reports, pill count, physician judgment, medication possession ratio based on pharmacy refill data, and electronic monitoring caps on all antiparkinsonian drugs [18, 19]. To the best of our knowledge, of the self-reported questionnaires, only the Morisky test has been thus far used among the PD patients population. Otherwise, the Morisky scale is the best known and the most widely used scale in the study of adherence, with several advantages: it identifies barriers to nonadherence, and it is short, easy to score, very adaptable to different groups of drugs in various diseases, populations, and countries. We opted to use the ARMS, which was found in previous studies as a reliable and valid instrument that can be used to evaluate adherence based on self-reporting by the chronically ill [17, 20]. As an example, among 429 patients with hypertension, those with low ARMS scores (indicating better adherence) were significantly more likely to have controlled diastolic blood pressure and tended to have both controlled systolic and total blood pressure [17]. This scale includes two distinct subscales: the 8-item medication-taking subscale assesses a patient's ability to correctly self-administer the prescribed regimen, and the 4-item prescription refill subscale assesses a patient's ability to refill medications on schedule. The ARMS correlated significantly with the Morisky adherence scale (Spearman's rho = −0.651, *p* < 0.01); moreover, it correlated more strongly with measures of refill adherence than did the Morisky scale [17]. Thus, the ARMS evaluates attitudes to adherence to medications and refills. So, unlike the Morisky scale, it brings information about refill adherence. This is important because medication taking and prescription refills are considered different medication use problems. Also, the ARMS is the first measure of adherence that demonstrates stability across patient literacy levels [17]. The scoring of the ARMS also differs from that of the Moryski scale. Contrary to Moryski, the ARMS does not provide ranges defining whether the patient's adherence is low or high, but higher scores assume lower adherence. In our study, 29 (25.9%) patients achieved the best possible minimum score, while 83 (74.1%) of patients reported lower adherence to the prescribed therapy. The ARMS has good positive predictive values helpful in identifying “true nonadherent patients” with a lower specificity to identify “false adherents” [21]. The disadvantage of both scales is that they focus on assessing the medication underuse part, yet the overuse of medications seems to be neglected in developing these instruments.

While the previous studies were inconsistent [18, 22], patient-related factors such as sex, age, and education level had no impact on our research. In a recent study [23], lower adherence was observed in males, in line with the data suggesting that females were more likely to be correct in their self-estimation of missed doses; however, men more accurately estimated their mistiming errors [24]. Adherence to therapy was influenced by aging-dependent comorbidities (i.e., increased prevalence of chronic diseases, cognitive decline, impaired vision, and hearing). Nevertheless, a category of elderly patients termed “young-old” were concerned about their health and showed better adherence than younger subjects [2, 7].

Most of the studies did not report the role of therapy-related factors on adherence, such as duration of therapy, frequency of intake, number of different medications per day, adverse events, and intake complexity [3, 22]. However, a large multicenter European study of adherence in PD that used electronic monitoring bottles showed that drugs given once daily had significantly better adherence when compared with medications prescribed more frequently [19]. In our study, no difference was found in the number of daily doses of levodopa and in the number of drugs the patients were taking between the two groups of patients, but the average daily dose of levodopa and the average value of LEDD were significantly higher in the group of patients with lower adherence than the adherent group. Such findings could be understood as a consequence and not a reason for poor adherence. The doctor must consider how the patient adheres to the therapy when assessing the patient's condition because unnecessary therapy adjustments may occur, such as increasing the doses or introducing additional drugs. Complications of dopaminergic therapy such as motor fluctuations were more frequent in a lower adherent group than the adherent group. Also, lower adherence was 2.8 times more likely among patients with motor fluctuations. Motor fluctuations are common during the disease course affecting 50% of patients treated with levodopa for 4–6 years [25]. They are caused by short levodopa half-life, erratic levodopa absorption, decreased dopamine striatal storage capacity in presynaptic vesicles, abnormal pulsatile stimulation of striatal dopamine receptors, and consequent hypersensitivity [26]. Finally, irregular medication intake may also contribute to motor fluctuations.

Among disease-related factors considered in this study (disease duration, severity of symptoms, presence of affective disorders, psychotic manifestations, and other NMSs) [3, 27, 28], we found that patients with lower therapy adherence had more severe disease, worse motor status, and poorer daily functioning. In a multicenter European study, poor medication adherence was significantly associated with poor motor scores (UPDRS III), more “off” time (UPDRS IV), and worse mobility. Furthermore, the overall burden of the NMS (NMSQuest total score) was associated with lower adherence, in line with the recent Straka et al.'s study [23]. Among NMSs, cardiovascular, apathy/attention-deficit/memory disorders, hallucinations/delusions, and symptoms such as changes in weight, excessive sweating, and diplopia were significantly more often represented in the lower adherence group. A few facts regarding NMSs are essential to mention: (1) NMSs are less responsive or nonresponsive to dopaminergic therapy compared to motor features, (2) NMSs responsive to levodopa can be a part of “off” time, (3) NMSs are often insufficiently recognized and therefore untreated, and patients rarely complain about them, (4) some of NMSs overlap with adverse effects of dopaminergic therapy, and (5) NMSs contribute significantly to the morbidity and strongly affect the QoL [29]. Taking all this into account, the insensitivity of the NMS to dopaminergic therapy is one possible explanation for irregular drug intake. Besides this, sometimes, a patient's expectations regarding the efficacy of therapy might be nonrealistic and could lead to the discontinuation of therapy. Erratic drug-taking in patients with early PD will not significantly affect the patient's functioning but will have a particular effect on patients in advanced PD, leading to a deterioration of both motor and nonmotor symptoms. In addition, dopaminergic medications can aggravate feeling unconscious, dizzy, or weak when getting up from a sitting or standing position and cause leg swelling. Also, dopaminergic drugs can induce confusion, hallucinations, and psychosis. Patients might stop taking medication to avoid such side effects in line with these data. Finally, although patients with the MoCA below 26 were excluded from this study, it cannot be ruled out the possibility that cognitive changes nevertheless contributed to lower adherence in this study since patients frequently reported attention and memory problems in NMSquest.

We found no difference in mean total HDRS scores between the two groups of patients. Still, in multivariate analysis, depression was the most potent independent risk factor, increasing the risk of low adherence 3.4 times. Many studies have shown that depression per se is a factor of low adherence not only among PD patients but also in the studies with patients suffering from other chronic diseases [28]: a meta-analysis of the effects of anxiety and depression on adherence had demonstrated that depressed patients were three times more likely to be nonadherent compared to nondepressed patients [30]. Possible explanations included (a) the fact that depression was often associated with hopelessness and lack of positive expectations and beliefs in the effectiveness of treatment, (b) social isolation and withdrawal might lead to a lack of support from the family and society in the patient's attempts to persist in treatment, and (c) cognitive decline and difficulties impaired abilities to follow treatment recommendations. Therefore, recognition and adequate treatment of depression might result in improved adherence. However, compared to previous inconsistent results [30], lower adherence was significantly associated with higher anxiety scores in our study.

This was the first study that used the ARMS scale for a neurological condition. ARMS scale is reliable and designed for use in patients with chronic diseases, and PD is an example of such disease. Also, this is the first scale that demonstrated stability across patient literacy levels [17]. This study has several limitations to be noted. First, the cross-sectional study design cannot fully establish whether a factor was the cause or consequence of the lower adherence. Second, medication adherence was measured through self-reporting, which can cause social desirability response bias. The concomitant use of both subjective and objective measures can provide higher reliability. Third, ARMS does not provide ranges that delineate the levels of patient adherence, and it has a lower specificity to identify “false adherents” [21]. Fourth, our sample size was relatively small, limiting statistical power. Finally, we excluded from the study all patients with MoCA below 26. Still, there is a possibility that some of our patients had coexistent cognitive impairment since MoCA is suitable for routine clinical screening of cognitive deficit and does not provide detailed cognitive status.

## 6. Conclusion

These findings suggest that lower adherence is common among PD patients. Identifying PD patients at risk of lower adherence is important in clinical settings to modify causes. Our results indicated that depressed PD patients compared to those without clinical depression have three times higher risk for lower adherence to pharmacotherapy. Recognition and adequate treatment of depression in PD could help improve adherence and, therefore, improve management and better clinical outcomes of PD patients. Also, PD NMS could contribute to lower adherence, which needs to be addressed in future studies.

## Figures and Tables

**Table 1:** Demographic and clinical characteristics of the study PD population (*N* = 112) and comparison of fully and lower adherent group.

Demographic and clinical characteristics and scores	All patients *N* = 112	Fully adherent *N* = 29	Lower adherent *N* = 83	*p* value
*Sex*				
Male^#^	65 (58.0)	16 (55.2)	49 (59.0)	NS
Female^#^	47 (42.0)	13 (44.8)	34 (41.0)	

Actual age (years)^*∗*^	67.0 ± 7.3	68.9 ± 8.2	66.4 ± 6.9	NS

*Education* ^*#*^				
Primary school	10 (8.9)	2 (6.9)	8 (9.6)	NS
Secondary school	69 (61.6)	16 (55.2)	53 (63.9)	
Faculty	33 (29.5)	11 (37.9)	22 (26.5)	

Age at onset (years)^*∗*^	59.2 ± 8.2	62.1 ± 8.8	58.1 ± 7.7	0.022
Disease duration (years)^*∗*^	7.9 ± 4.5	6.8 ± 3.7	8.4 ± 4.6	NS
*Form of the disease* ^*#*^				
Tremor-predominant	40 (35.7)	12 (41.4)	28 (33.7)	NS
Akinetic-rigid	72 (64.3)	17 (58.6)	55 (66.3)	

Number of levodopa daily doses^*∗*^	4.6 ± 0.9	4.4 ± 0.9	4.7 ± 0.9	NS
Number of drugs per day^*∗∗*^	3.3 ± 1.4	3.0 ± 1.3	3.5 ± 1.4	NS
Levodopa average dose (mg/day)^*∗*^	537.0 ± 214.0	462.1 ± 177.1	564.6 ± 220.7	0.018
LEED (mg/day)^*∗*^	853.8 ± 361.8	702.8 ± 307.4	906.5 ± 366.1	0.014
Patients with motor fluctuations^#^	77 (68.8)	15 (51.7)	62 (74.7)	0.022
Patients with dyskinesias^#^	51 (45.5)	9 (31.0)	42 (50.6)	NS
Patients with hallucinations^#^	39 (34.8)	5 (17.2)	34 (41.0)	0.021
UPDRS total score^*∗*^	56.3 ± 20.8	49.6 ± 22.3	58.7 ± 19.9	0.013
UPDRS I^*∗*^	3.8 ± 2.5	3.1 ± 2.5	4.0 ± 2.4	NS
UPDRS II^*∗*^	14.5 ± 6.4	12.8 ± 6.9	15.1 ± 6.3	NS
UPDRS III^*∗*^	33.7 ± 11.5	30.2 ± 13.3	34.9 ± 10.6	0.017
UPDRS IV^*∗*^	4.3 ± 3.7	3.3 ± 3.5	4.7 ± 3.7	0.041
HY stage^*∗*^	2.5 ± 0.6	2.4 ± 0.6	2.6 ± 0.6	NS
S&E scale^*∗*^	75.6 ± 13.8	80.3 ± 12.7	73.9 ± 13.9	0.032
HDRS total score^*∗*^	10.9 ± 7.3	8.9 ± 7.1	11.7 ± 7.2	NS
HARS total score^*∗*^	8.9 ± 6.4	7.3 ± 6.9	9.6 ± 6.1	0.043
NMSQuest^*∗*^	10.9 ± 5.7	8.9 ± 4.6	11.7 ± 5.9	0.023

^#^Values are the number of patients (percentage); ^*∗*^Values are mean ± standard deviation; NS = not significant. ^∗^indicates the number of drugs (antiparkinsonian drugs, benzodiazepines, antipsychotics, and antidepressants) per day. *p* values from Mann–Whitney *U* tests for continuous variables and the Pearson chi-squared test for categorical variables comparing fully and lower adherent groups are presented. The statistically significant difference is *p* < 0.05. Patient characteristics that differed significantly between groups (*p* < 0.05) are shown in boldface. LEDD, levodopa equivalent daily dose; UPDRS, Unified Parkinson's Disease Rating Score; HY, Hoehn and Yahr stage; S&E, Schwab–England Scale; HDRS, Hamilton Depression Rating Scale; HARS, Hamilton Anxiety Rating Scale; NMSQuest, Non-Motor Symptom Questionnaire.

**Table 2:** Variables associated with lower adherence on medication and logistic regression model examining predictors of lower adherence.

Variables	B	OR (95% CI for OR)	*p* value	B	OR (95% CI for OR)	*p* value
Sex	−0.158	0.854 (0.364–2.003)	0.717			
Actual age	−0.049	0.952 (0.895–1.012)	0.117			
Education	−0.421	0.657 (0.314–1.373)	0.264			
Age at onset	**−0.064**	**0.938 (0.887–0.992)**	**0.026**			0.195
Disease duration (years)	0.096	1.101 (0.981–1.235)	0.101			
Form of disease	0.327	1.387 (0.582–3.302)	0.460			
Number of levodopa doses per day	0.319	1.375 (0.808–2.339)	0.240			
Number of drugs per day^∗^	0.267	1.306 (0.938–1.818)	0.113			
Levodopa average daily dose (mg/day)	**0.002**	**1.002 (1.000–1.005)**	**0.031**			0.064
LEDD (mg/day)	**0.002**	**1.002 (1.000–1.003)**	**0.011**			0.107
Motor fluctuations	**1.014**	**2.756 (1.142–6.647)**	**0.024**			0.236
Presence of dyskinesia	0.823	2.276 (0.929–5.581)	0.072			
Presence of hallucinations	**1.203**	**3.331 (1.156–9.596)**	**0.026**			0.116
UPDRS total score	**0.025**	**1.026 (1.001–1.052)**	**0.044**			0.493
HY stage	0.701	2.016 (0.893–4.553)	0.091			
S&E scale	**−0.038**	**0.962 (0.928–0.998)**	**0.036**			0.337
HDRS>7	**1.133**	**3.106 (1.298–7.432)**	**0.011**	**1.238**	**3.449 (1.425-8,350)**	**0.006**
HARS>14	0.212	1.236 (0.411–3.719)	0.706			
NMSQuest	**0.093**	**1.097 (1.012–1.189)**	**0.025**			0.459

^∗^indicates the number of drugs per day (number of antiparkinsonian drugs, benzodiazepines, antipsychotics, and antidepressants per day). Patient characteristics that differed significantly between groups (*p* < 0.05) are shown in boldface. B, B coefficient; OR, odd ratio; CI, confidence interval; LEDD, levodopa equivalent daily dose; UPDRS, Unified Parkinson's Disease Rating Score; HY, Hoehn and Yahr stage; S&E, Schwab–England scale; HDRS, Hamilton Depression Rating Scale; HARS, Hamilton Anxiety Rating Scale; NMSQuest, Non-Motor Symptom Questionnaire.

## Data Availability

The datasets generated during and/or analyzed during the current study are available from the corresponding author on reasonable request.

## Additional Points

*What is Known?* Pharmacological management of PD is complex, which may negatively impact patients' adherence to therapy. Irregular medication intake is associated with the progressive vanishing of therapeutic effect resulting in motor fluctuations and dyskinesias, and it also could cause a worsening of symptoms. The incidence of suboptimal adherence in PD varies between 10% and 67%. Recognizing factors that affect adherence may help achieve better outcomes for PD patients. *What is New?* This is the first study that used the ARMS in a group of PD patients. Unlike other self-reported questionnaires, in addition to attitudes to medication adherence, ARMS provides information about refill adherence. Also, this scale demonstrates stability across patient literacy levels. This is the first study that examined adherence to antiparkinsonian medications among Serbian PD patients. Our study has confirmed the role of depression in nonadherence and suggested the possible impact of other nonmotor symptoms of PD as potential contributing factors to poorer adherence that should be examined in detail in future studies.
